# Recent Advances of Biomedical Materials for Prevention of Post-ESD Esophageal Stricture

**DOI:** 10.3389/fbioe.2021.792929

**Published:** 2021-12-22

**Authors:** Yuchen Bao, Zhenguang Li, Yingze Li, Tao Chen, Yu Cheng, Meidong Xu

**Affiliations:** ^1^ Translational Medical Center for Stem Cell Therapy and Institute for Regenerative Medicine, Institute for Translational Nanomedicine, Shanghai East Hospital, Tongji University School of Medicine, Shanghai, China; ^2^ Endoscopy Center, Shanghai East Hospital, Tongji University School of Medicine, Shanghai, China

**Keywords:** endoscopic submucosal dissection (ESD), esophageal stricture, tissue engineering, biomedical polymer, biomedical derived materials

## Abstract

Esophageal stricture commonly occurs in patients that have suffered from endoscopic submucosal dissection (ESD), and it makes swallowing difficult for patients, significantly reducing their life qualities. So far, the prevention strategies applied in clinical practice for post-ESD esophageal stricture usually bring various inevitable complications, which drastically counteract their effectiveness. Nowadays, with the widespread investigation and application of biomedical materials, lots of novel approaches have been devised in terms of the prevention of esophageal stricture. Biomedical polymers and biomedical-derived materials are the most used biomedical materials to prevent esophageal stricture after ESD. Both of biomedical polymers and biomedical-derived materials possess great physicochemical properties such as biocompatibility and biodegradability. Moreover, some biomedical polymers can be used as scaffolds to promote cell growth, and biomedical-derived materials have biological functions similar to natural organisms, so they are important in tissue engineering. In this review, we have summarized the current approaches for preventing esophageal stricture and put emphasis on the discussion of the roles biomedical polymers and biomedical-derived materials acted in esophageal stricture prevention. Meanwhile, we proposed several potential methods that may be highly rational and feasible in esophageal stricture prevention based on other researches associated with biomedical materials. This review is expected to offer a significant inspiration from biomedical materials to explore more effective, safer, and more economical strategies to manage post-ESD esophageal stricture.

## Introduction

Esophageal cancer, a malignant tumor occurring in the esophagus, has the seventh highest incidence and the sixth highest mortality rate worldwide (Sung et al., 2021). Although the overall 5-year survival of malignant esophageal cancer is less than 20% in Asian people, early esophageal cancer patients can obtain good prognosis if merely the mucosal layer or superficial submucosal layer is invaded (Siegel et al., 2017). With the remarkable development of endoscopic technology, endoscopic submucosal dissection (ESD) has been acknowledged as the standard therapy in clinics for early esophageal cancer because of minimally invasive tumor excision for minimizing the risk of deterioration (Shah and Gerdes 2015). The ESD employs high-frequency electrosurgical knife and special equipment under the endoscope to gradually peel off the gastrointestinal lesion from the normal submucosa to achieve complete resection of the lesions, reducing local recurrence and metastasis (Fujishiro et al., 2009; Mochizuki et al., 2012). Meanwhile, ESD shows optimistic results in the evaluation of postoperative efficacy. It is recorded that the *en bloc* resection rate and complete resection rate of esophageal ESD is 90%–100% and 90%–97.4%, respectively (Takahashi et al., 2010; Nishizawa and Suzuki 2020). In general, ESD is an economical, safe, and reliable method to remove superficial lesions of the digestive tract.

However, there are still some complications that occurred during or after ESD, such as bleeding, perforation, and stricture. Esophageal stricture, which presents as a significant tortuosity of the esophageal lumen and is difficult to pass through by conventional gastroscopy, is a particularly common and serious postoperative complication (Lew and Kochman 2002; Ono et al., 2009), significantly reducing the living quality of patients for its serious consequences such as reflux and inhalation pneumonia. The incidence of post-operative esophageal stricture would reach 90% if patients suffer from mucosal defects over three-quarters of the circumferential of the esophagus (Nishizawa and Suzuki 2020). Unlike other complications such as bleeding and perforation, skilled operation is not able to fundamentally prevent the occurrence of esophageal stricture.

**FIGURE 1:**
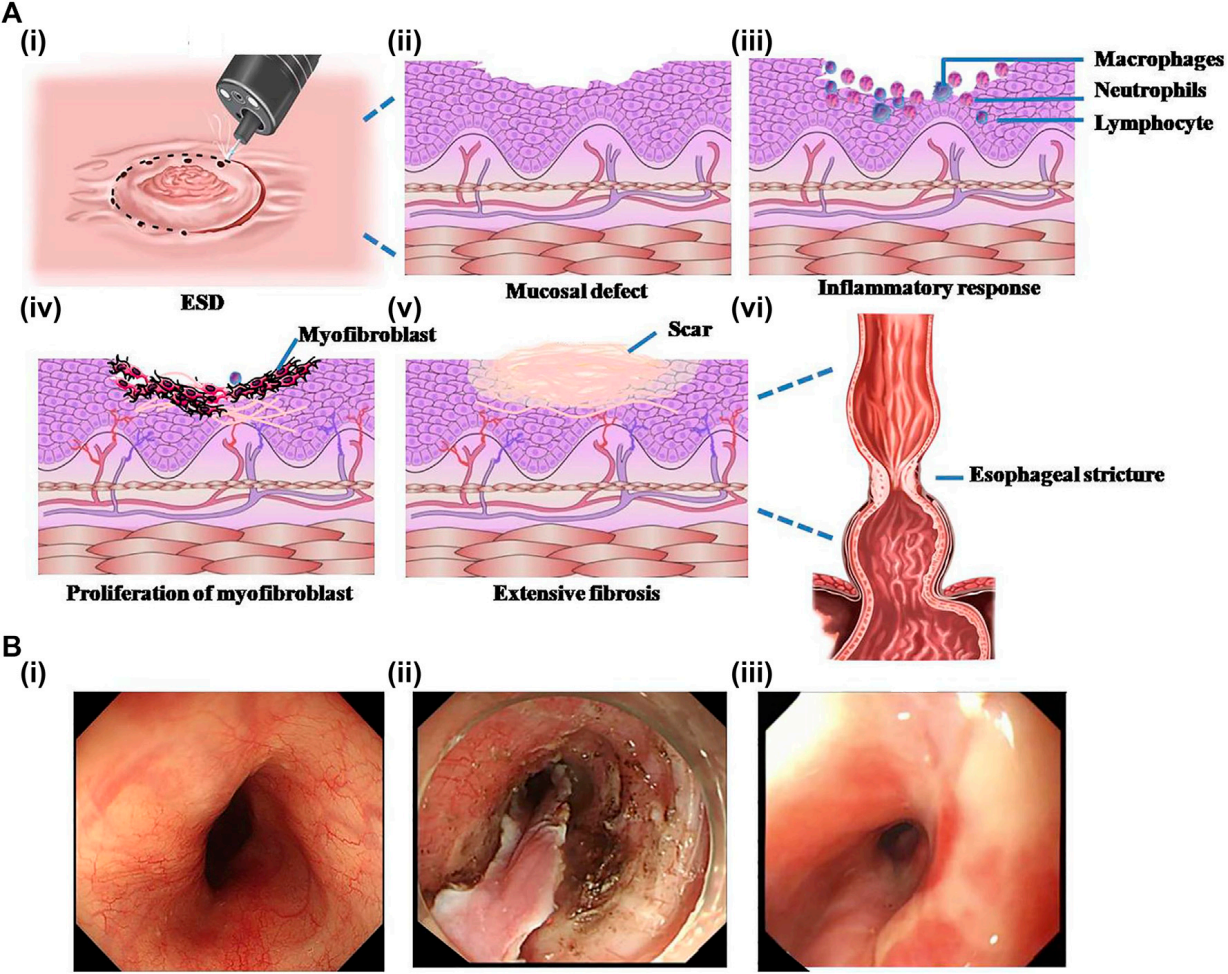
Schematic representation of the formation process of esophageal stricture after endoscopic submucosal dissection (ESD) and the endoscopic photographs of esophageal stricture. **(A)** The schematic diagram of the formation of esophageal stricture. **(i)** ESD surgery. **(ii)** Irregular mucosal defect left after surgery. **(iii)** Acute inflammatory reaction in exposed mucosal wounds. **(iv)** Massive proliferation of myofibroblasts after inflammation accompanied by angiogenesis in the submucosa. **(v)** Extensive fibrosis of the esophageal mucosal wound forming a scar. **(vi)** Scar formation and contracture at the wound result in esophageal stricture. **(B)** Endoscopic pictures of the esophagus. **(i)** Normal esophagus. **(ii)** Esophagus with mucosal layer stripped by ESD. **(iii)** Stricture-forming esophagus. Reproduced from Chu et al. (2019). Copyright (2019), with permission from Springer Nature.

It has been found that esophageal stricture usually occurs within 2–4 weeks after mucosal resection (Mizuta et al., 2009; Ono et al., 2009). Though the specific mechanism of esophageal stricture after ESD has not been completely illuminated, many experiments have indicated that mucosal defect is the initial and most important condition in the process of esophageal stricture (Honda et al., 2010; Pech et al., 2014). Although it has been confirmed that the wound over three-quarters of the esophageal circumference after ESD would cause obvious esophageal stricture (Ono et al., 2009; Chu et al., 2019), the longitudinal length of the esophageal mucosal defect and the depth of the lesions are also reliable risk factors for the occurrence of postoperative stricture (Alvarez Herrero et al., 2011). The esophageal mucosal defect caused by ESD will promote inflammatory response and subsequently enter into the staggered and complicated wound-repairing process (Radu et al., 2004), which can be simply divided into three phases: inflammatory response, epithelial proliferation, and extracellular matrix remodeling (Figure 1) (Broughton et al., 2006). The wound-healing process after endoscopic mucosal resection (EMR) has been observed in an animal experiment conducted by Honda et al. (2010). The inflammation at defect lesions disappeared after 1 week of operation, and then new blood vessels and fibrous tissues proliferated significantly, accompanied by epithelial cells growing and migrating from the edge of the lesions. More than 1 month after EMR, the mucosal defect was gradually covered by squamous epithelium, and the submucosa was replaced by large and dense collagen fibers. Meanwhile, the muscle fibers of the muscularis propria gradually atrophied and eventually became fibrotic, reducing the elasticity and compliance of the esophageal wall. Besides, the massive proliferation of collagen fibers and extensive fibrosis can form scarring tissues, resulting in further esophageal stricture. Therefore, effective prevention methods of esophageal stricture can be approached from the following aspects: inhibiting initial inflammatory response, promoting epithelial regeneration, and inhibiting fibrosis.

The main methods currently used in clinics for treating or preventing post-ESD esophageal stricture include balloon dilation, stent dilation, and pharmaceutical prophylaxis. Although these methods are effective to some extent, they also bring certain different extents of complications, which seriously affect the physical and mental health of patients. Thus, it is essential to optimize and explore new methods for the prevention of esophageal stricture. In recent decades, tissue engineering has been considered as the most potential technology for regenerative medicine, in which biomedical materials are employed as scaffold to support cell migration, adhesion, proliferation, and differentiation due to their unique properties including good biocompatibility and biodegradability, stable chemical properties, and mechanical properties matching the tissues. In the application of esophageal stricture prevention after ESD, biomedical materials derived from polymers and natural tissues can be used as tissue engineering scaffold to support mucosal epithelial cell adhesion and proliferation and promote esophageal mucosal repairing.

**FIGURE 2:**
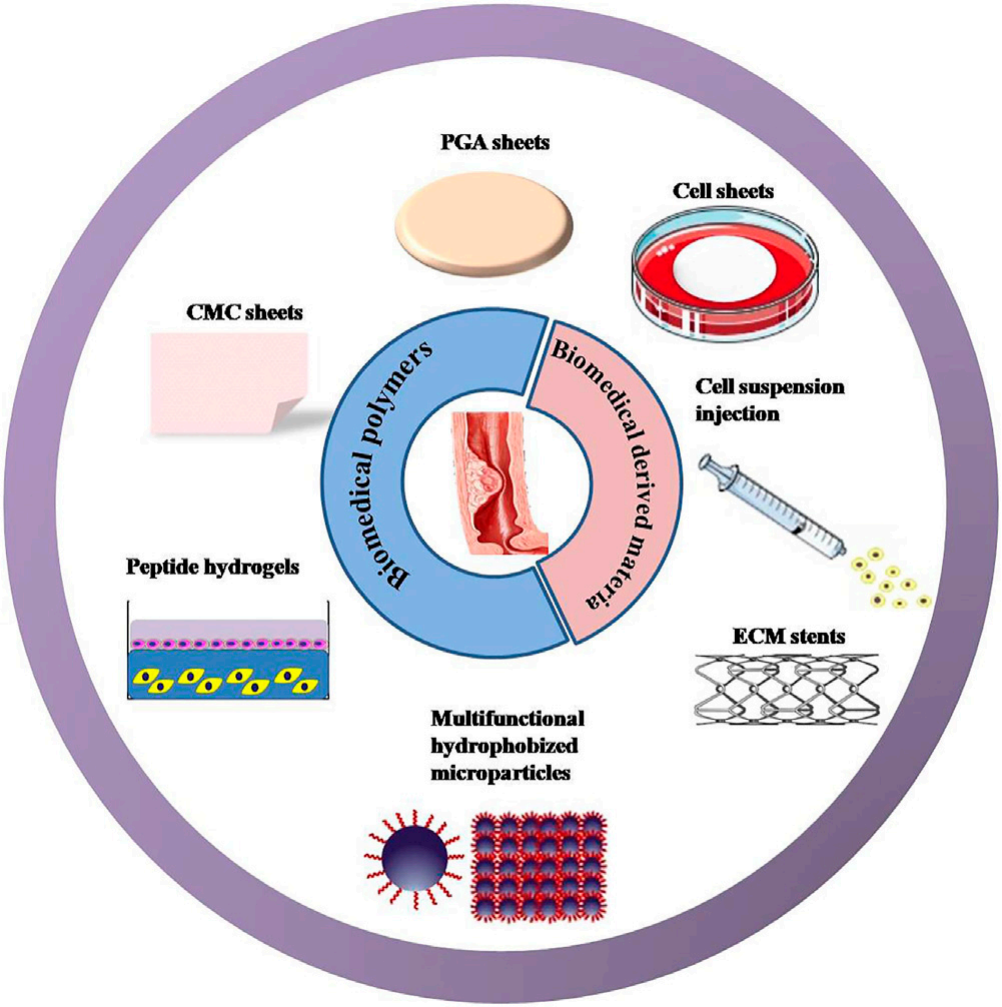
New strategies that have been reported to prevent post-ESD esophageal stricture. Approaches involve biomedical polymers: including polyglycolic acid (PGA)/carboxymethyl cellulose (CMC) sheets, peptide hydrogels, and multifunctional hydrophobized microparticles. Methods covering the biomedical-derived materials incorporating cell sheets, cell suspension, and extracellular matrix (ECM) stents.

In this review, we are going to integrate approaches involving biomedical materials that have been reported for the management of esophageal stricture (Figure 2). Furthermore, novel preventive strategies are proposed based on the extensive investigations and applications of biocompatible and biodegradable polymers. These proposed innovations may be reasonable and practicable by inhibiting inflammatory response, promoting epithelial regeneration, or reducing excessive fibrosis on the base of intervention to the formation of esophageal stricture. As an ultimate aim of the review, we hope the comprehensive analysis of these strategies and in-depth thinking will provide a strong reference value for engineering more effective and safe systems to drastically reduce the incidence of esophageal stricture after ESD.

## Current Clinical Approaches for the Prevention of Post-ESD Esophageal Stricture

Up to now, many methods of preventing or treating esophageal stricture have been widely applied in clinical practice, including pharmacological treatments and mechanical manipulation, such as endoscopic dilation and endoscopic stent implantation (Ezoe et al., 2011; Yamaguchi et al., 2011; Machida et al., 2012; Ye et al., 2016). However, these methods bring certain different extents of complications. Endoscopic balloon dilatation (EBD) usually needs to be underwent multiple times to prevent re-stricture and may result in bleeding, perforation, bacteremia, and other complications, which are time-consuming and bring great pain to patients. Endoscopic stents mainly include metal stents and biodegradable stents. Metal stents have a certain preventive effect on stricture after ESD, but they may cause complications such as stent displacement, gastrointestinal bleeding, perforation, and granulation hyperplasia (Bakken et al., 2010). Contrastively, biodegradable stents represented by polylactic acid show good biocompatibility and ideal degradability, while it has poor capacity of self-expansion and weak mechanical strength, accompanied by complicated placement procedure. Saito et al. (2008) reported that the stent would dislocate in 10–21 days with the biodegradation of polylactic acid, while the effectiveness of stents in preventing stricture in such a short period of time is doubtful. Steroids are common anti-inflammatory drugs, and they are widely used in clinical practice to inhibit inflammatory response (Rhen and Cidlowski 2005; Mori et al., 2013). However, the use of systemic steroids is frequently accompanied by adverse reactions such as immunosuppression, osteoporosis, and other hormone-induced diseases. Even local injection of steroids could also bring complications related to puncture operations, such as esophageal perforation, esophageal abscess, mediastinal abscess, pleural effusion, and gastrointestinal bleeding. What is worse, steroids are poorly utilized in local injection (Rajan et al., 2005; Lee et al., 2013).

## Biomedical Polymers

Biomedical polymers used for esophageal stricture prevention can be categorized as synthetic polymers and naturally derived polymers according to their material sources. Synthetic polymers are artificially synthesized by chemical or physical means. Naturally derived polymers are made from natural polymers produced by living organisms with appropriate modifications. Naturally derived polymers have better biocompatibility and biodegradability than synthetic polymers.

### Synthetic Polymers

#### Polyglycolic Acid Sheets

**FIGURE 3:**
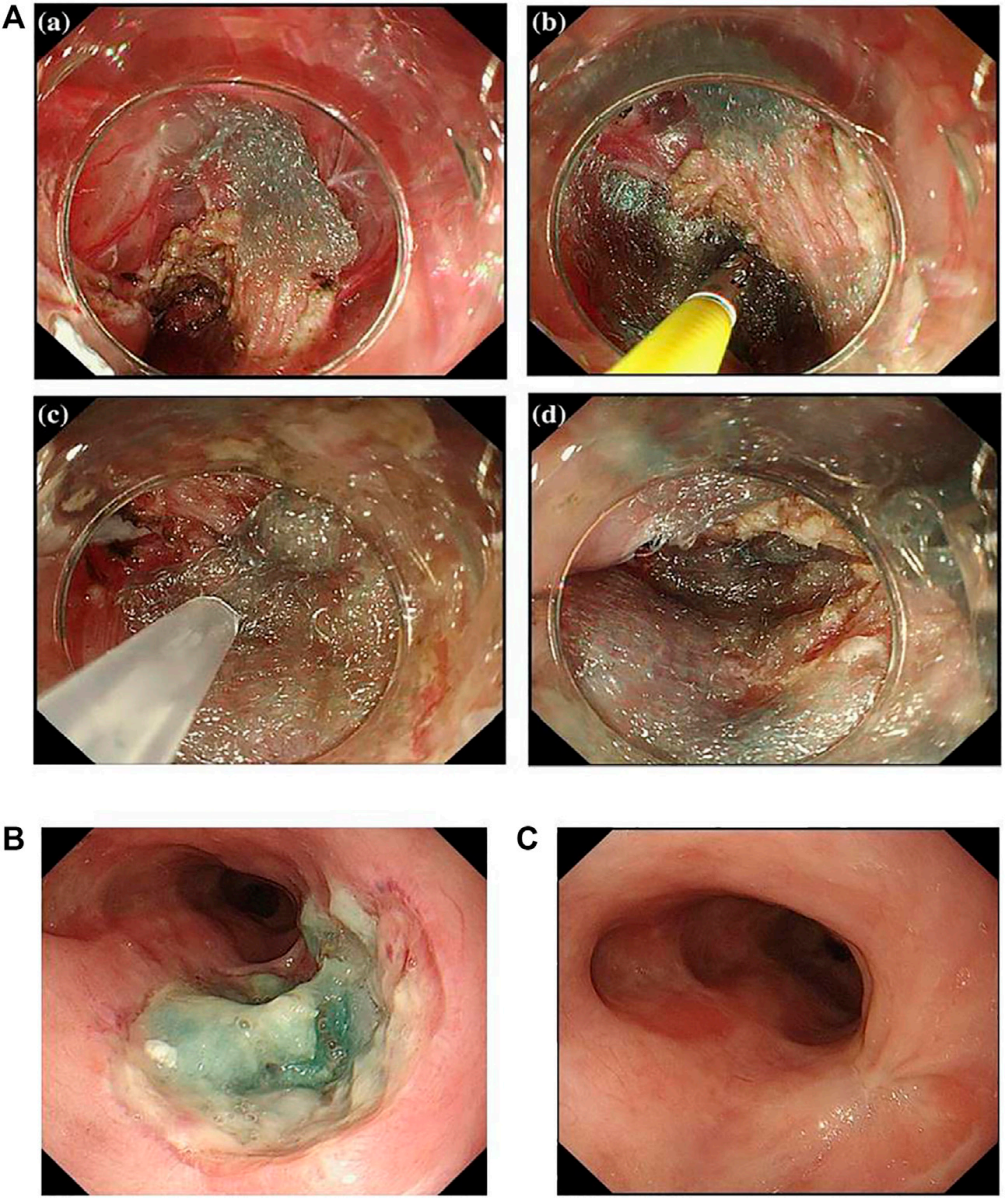
Newly proposed healing dressings based on biocompatible biomedical polymers. **(A)** Esophageal perforation was completely covered by PGA sheets. **(B)** The endoscopic image of the esophageal perforation with PGA sheets after 6 days of ESD. **(C)** The endoscopic image of the esophagus after 3 months of ESD. Reproduced from Seehawong et al. (2019). Copyright (2019), with permission from Springer Nature.

Polyglycolic acid (PGA) sheets are biodegradable fabric dressings made of polyglycolic acid. They are biocompatible and can be fully absorbed within 5 months. PGA is the first biomedical synthetic polymer used in clinical medicine, and they have a high degree of crystallinity to make a large tensile elastic modulus as well as excellent mechanical properties (Budak et al., 2020). Therefore, PGA and its derivatives have been employed in medical fields such as drug delivery and dental and orthopedic systems (Takimoto et al., 2014; Sakaguchi et al., 2016; Sharma and Sharma 2018), and PGA sheets, as a form of tissue engineering scaffold, are widely used in gastrointestinal field. In Japan, PGA sheets have been used to promote wound healing after resection of oral tumors (Inokuchi et al., 2017). In the recent decade, PGA sheets (Neoveil; Gunze Co., Kyoto, Japan) are applied in endoscopic technology for the closure of perforations and postoperative homeostasis. Seehawong et al. (2019) used PGA sheets combined with fibrin glue to treat esophageal perforation after ESD (Figure 3A). They found that PGA sheets and fibrin glue facilitated the regeneration of mucosal tissues after ESD in a 74-year-old female, and perforations were all closed by PGA sheets without obvious leakage after 6 days of operation (Figure 3B). After 3 months of ESD, the esophageal ulcer repaired completely without formation of esophageal stricture (Figure 3C). Sakaguchi et al. (2015) conducted a research in which 11 patients received submucosal injection of steroids immediately after esophageal ESD, and then PGA was shielded on the esophageal defect. At last, they found that the incidence of esophageal stricture was reduced without requirement for EBD (Sakaguchi et al., 2015). Chai et al. (2018) found that the combination of PGA and stent dilation could significantly suppress the esophageal stricture compared to using individual stent in 70 patients (incidence was 20.5%:46.9%). To sum up, PGA sheets combined with fibrin glue have been proved to be effective to prevent post-ESD stricture. Though PGA has many advantages in the prevention of esophageal stricture, it is difficult for them to stay at the defect site for a long time. In some circumstances, some patients may be allergic to the degradation products of PGA. Besides, the risk of infection and bleeding during the application still needs to be discussed.

#### Peptide Hydrogels

**FIGURE 4:**
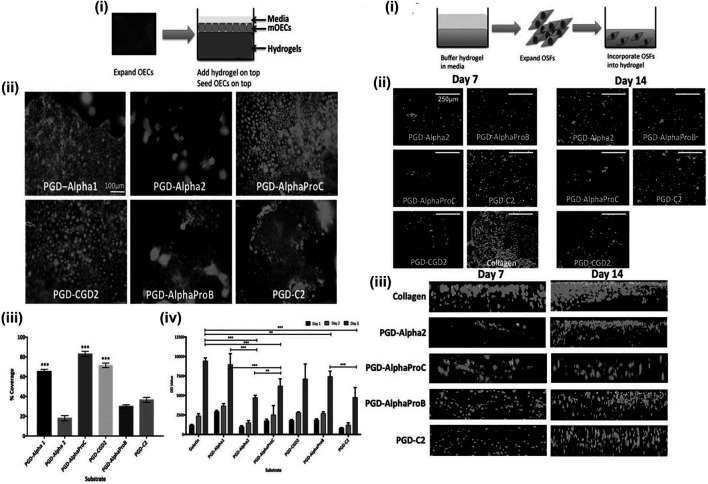
The behaviors of mouse esophageal epithelial cells (mOECs) and rat esophageal stromal fibroblasts (rOSFs) cultured on the surface of peptide hydrogels **(A)** and embedded within peptide hydrogels **(B)**. **[A (i)]** Schematic diagram of mOECs cultured on the surface of peptide hydrogels. **[A (ii)]** The viability and proliferation of mOECs cultured on different peptide hydrogel surfaces for 3 days. **[A (iii, iv)]** The assessment of metabolic viability of mOECs cultured on the surface of different peptide hydrogels at different time points. **[B (i)]** Schematic diagram of rOSFs cultured into peptide hydrogels. **[B (ii)]** The viability and proliferation of rOSFs cultured into different peptide hydrogels. **[B (iii)]** The distribution of rOSFs within the different peptide hydrogels at the culture time point of 7 and 14 days. Reproduced from Kumar et al. (2017). Copyright (2017), with permission from John Wiley and Sons.

Peptide hydrogel is a kind of hydrogel formed by cross-linked polymer chains of polypeptides or polypeptide derivatives (Zhou et al., 2009). It can be classified as chemical cross-linked peptide hydrogel and physical peptide hydrogel according to the formation type (Jonker et al., 2012). Physical peptide hydrogels are made by the intra- and intermolecular self-assembly, which derived from non-covalent interactions, including hydrogen bonding, electrostatic interactions, hydrophobic interactions, and so on (Tsonchev et al., 2004; Paramonov et al., 2006; de Groot et al., 2007; Bowerman et al., 2011; Koutsopoulos 2016). Therefore, physical peptide hydrogels are also considered to be self-assembling peptide hydrogels (SAPHs). Because self-assembling peptide hydrogels are formed gradually from molecules to nanofibers and then to the final network, the preparation process has a strong influence on the structure and properties of SAPHs. The peptide constituent gives SAPHs excellent properties of biocompatibility and biodegradability. Moreover, the preparation of SAPHs does not involve crosslinking reagents or organic solvents virtually, which makes SAPHs one of the promising options for biomedical applications, and SAPHs are injectable owing to the shear thinning property (Bakota et al., 2011; Lian et al., 2016; Zhang et al., 2017; Nguyen et al., 2018; Zhang et al., 2018; Gong et al., 2019). Besides, the structure of SAPHs is similar to the extracellular matrix, so they can be used as a scaffold in cell culture and tissue engineering, indicating a great potential of SAPHs in promoting repair of esophageal defects after ESD (Wan et al., 2016). Therefore, synthetic peptide hydrogels can be a disposal for preventing esophageal stricture in some way. Kumar et al. (2017) purchased a library of synthetic SAPHs from a company. Each hydrogel was different in terms of peptide sequence, stiffness, and overall charge (Kumar et al., 2017). These synthetic SAPHs supported bioactivities and functions of esophageal cells, realizing epithelialization and stratification during *in vitro* three-dimensional co-culture (Figure 4). In their study, mouse esophageal epithelial cells (mOECs) were seeded on the surface of peptide hydrogels (Figure 4A), while rat esophageal stromal fibroblasts (rOSFs) were incorporated into peptide hydrogels (Figure 4A, B). After a series of experimental evaluations, they found that the behaviors of mOECs such as morphology, proliferation, the formation of epithelial cell layers, and migration activity were influenced by distinctive properties of different hydrogels (Figure 4A–D). Similarly, the stiffness, charge, and mechanical properties of peptide hydrogels also affected responses of rOSFs (Figure 4B–D). The optimal composite hydrogel systems for 3D co-culture were favorable to both cell types and could successfully support the formation of a functional, uninterrupted epithelial sheet within a few days of incubation. However, the study was conducted merely at the cellular level, so the preventive effect and safety of these synthetic SAPHs need to be further studied in in-depth research.

### Naturally Derived Polymers

#### Carboxymethyl Cellulose Sheets

**FIGURE 5:**
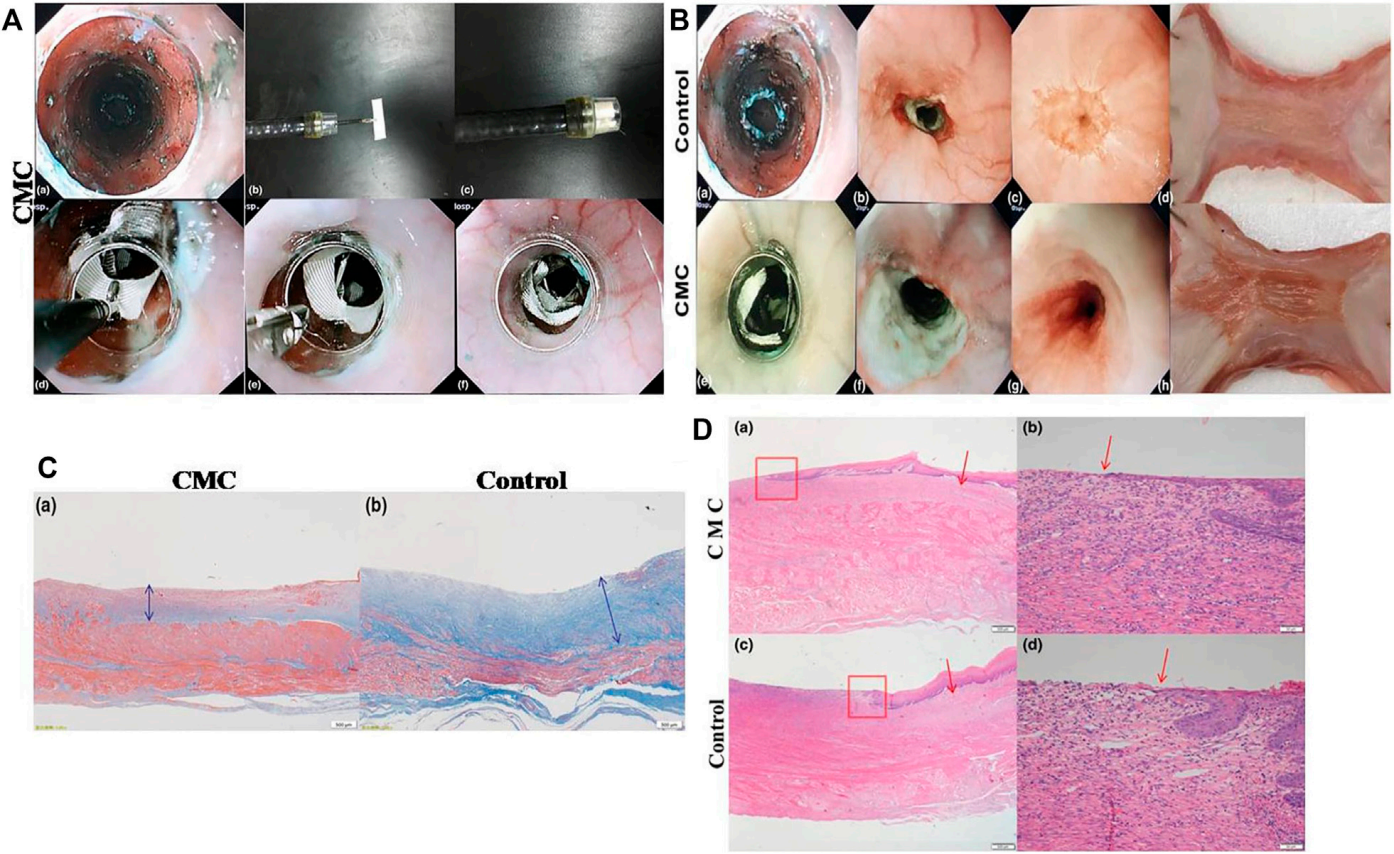
The application of CMC sheet to prevent esophageal stricture after ESD. **(A)** The process of delivering CMC sheet to the defect of the esophagus. **(B)** The comparison of the esophagus between the control group and the CMC group. **(C)** The comparison of fibrosis thickness (blue double-headed arrow) in the submucosa of the esophagus between the CMC-treated group and the control group. **(D)** The comparison of regenerated epithelial lengths of the esophagus between the CMC-treated group and the control group (red boxes designate the boundary between the regenerated mucosal layer and original mucosa, and red arrows refer to the edge of the regenerating mucosal epithelium). The esophageal mucosal epithelium regeneration in the CMC group was better than that in control group. Reproduced from Tang et al. (2018b). Copyright (2018), with permission from John Wiley and Sons.

Cellulose is a plant-derived polymer, and it is a renewably abundant resource in nature. Carboxymethyl cellulose (CMC) is obtained by carboxymethylation of cellulose. CMC sheets are biocompatible and biodegradable suture materials composed of modified hyaluronic acid and CMC, and they are harmless to people. Up to now, some clinical studies have demonstrated the effect of CMC sheets in wound healing (Bristow and Montz 2005; Huang et al., 2013). Tang et al. (2018b) covered small CMC sheets on the mucosal defects of the esophagus after ESD in pigs, and they found that the incidence of esophageal stricture in the CMC sheet treatment group was lower than that in the control group (71.4% vs 100%), with better food tolerance in the CMC sheet group (Figure 5). In the study, they delivered a CMC sheet above the mucosal defect by biopsy forceps to cover the defect fully in pigs (Figure 5A). Two weeks later, the pigs were killed, and the esophagus of each pig was excised and cultured to observe and evaluate esophageal stricture. As shown in Figure 5B, after 2 weeks of the implementation of ESD, severe esophageal stricture was seen in the control group, while the esophagus with treatment of CMC only showed slight stenosis. After histological evaluation of the esophagus in the CMC treatment and control groups, it was found that the fibrosis degree in the submucosa of the esophagus in the control group was significantly higher than that in the CMC-treated group (Figure 5C). They suggested that CMC sheets had an anti-fibrosis effect by regulating the expression of transforming growth factor beta (TGF-β1), which is a kind of growth factor associated with fibrosis (Sporn and Roberts 1989). Besides, the mucosal regeneration of the esophagus in the CMC-treated group was better than that in the control group (Figure 5D). Similar to PGA sheets, CMC sheets could play the role of biophysical barriers to protect the wound. The essence of mucopolysaccharides possessed by CMC allows them to adhere to the mucosal defect within minutes after being exposed to moisture. Gago et al. (2003) made a comparative cell experiment and concluded that the ability of CMC sheets to reduce fibrosis probably stems from its function as a physical barrier, though the exact mechanism has not been elucidated. These studies proved that CMC sheets have a certain effect on preventing esophageal stricture with safety after ESD. However, researchers discover that CMC sheets formed barriers for approximately 7 days but no more than 14 days due to their good biodegradability, and the short duration of action of CMC sheets can weaken the fibrosis inhibition effect. Besides, CMC sheets cannot achieve a strong and long adhesion to tissues. Last but not the least, a short-term study may be insufficient for elucidating the mechanism of CMC sheets to suppress the wound fibrosis and evaluating the effect on preventing esophageal stricture. Furthermore, larger samples are necessary and more basic research need to be done to demonstrate the efficacy and mechanism of action of CMC sheets before clinical application.

#### Multifunctional Colloidal Dressing

**FIGURE 6:**
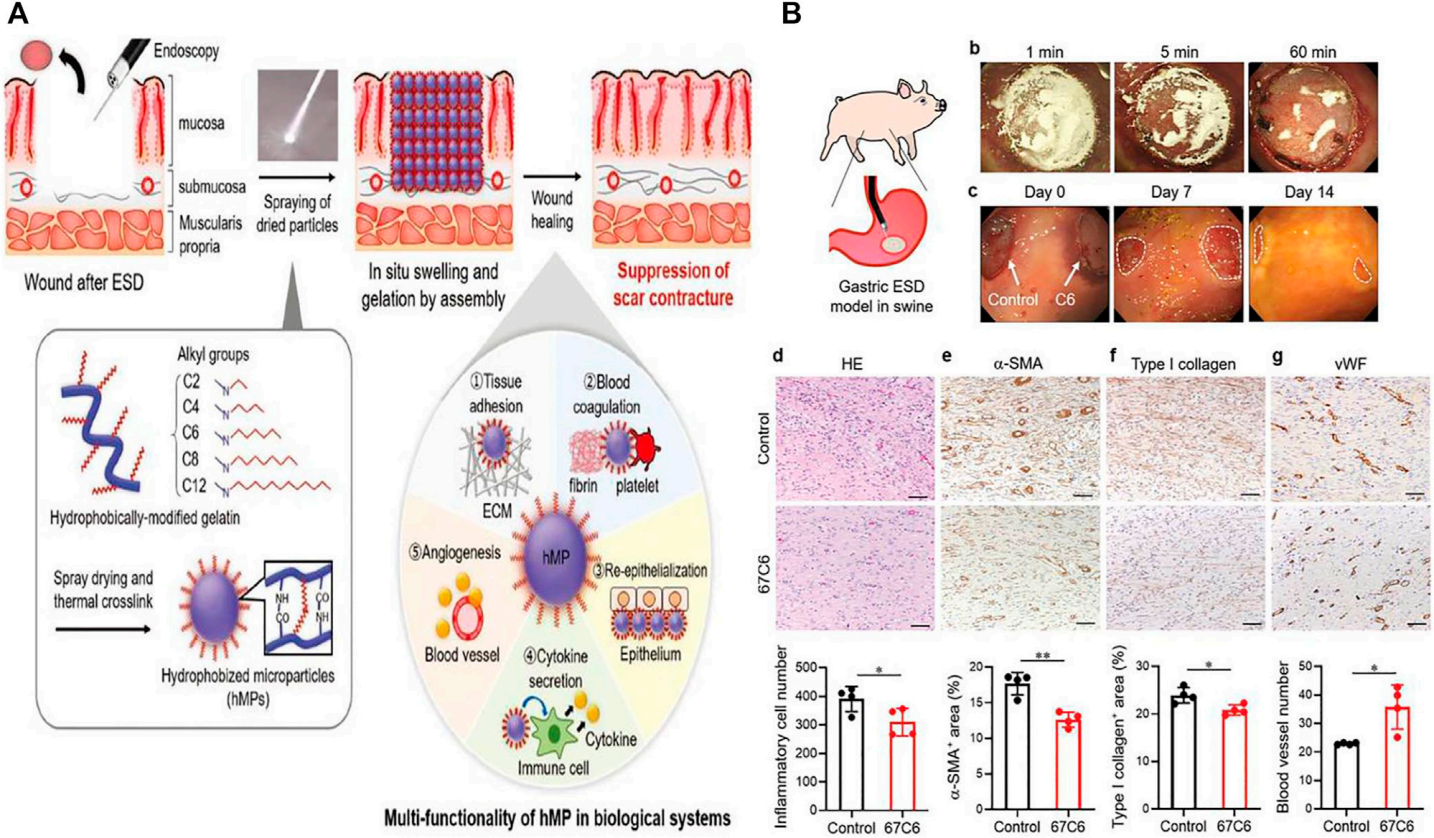
The preparation and application of colloidal wound dressings to facilitate ulcer healing after ESD. **(A)** The schematic diagram of multifunctional hydrophobic colloidal dressings for wound repair. **(B)** Hydrophobic colloidal dressings effectively suppressed wound fibrosis after ESD in the swine stomach model. Reproduced from Nishiguchi et al. (2019). Copyright (2019), with permission from John Wiley and Sons.

A multifunctional colloidal dressing was prepared by Nishiguchi et al. (2019) to accelerate wound healing after ESD (Figure 6). The wound dressing was composed of hydrophobic microparticles (hMPs), which were prepared as follows: gelatin (Gltn), which is produced by the partial hydrolysis of collagen from the organism, was modified with aliphatic aldehyde to synthesize hydrophobically modified Gltn (hm-Gltn), and then, granulation of hm-Gltn was realized using the spray drying method before being formed into dried hMPs by thermal crosslinking route. The dried hMPs swelled in esophageal exudates when they were sprayed to the artificial defects, and aggregation of hMPs formed a hydrogel layer on the surface of post-ESD wound to enter the subsequent treatment cycle (Figure 6A). Multi-functionality of hMPs under wet environments based on hydrophobic interaction included tissue adhesiveness, acceleration of blood coagulation, enhanced epithelialization, controlled inflammation, and enhanced angiogenesis. The adhesion strength of colloidal dressing substantially enhances as the alkyl chain length of aliphatic aldehyde increases at a certain extent. While the balance between hydrophobicity and hydrophilicity of hMPs is critical for achieving strong adhesion to live tissues, hydrophobically modified polymers can elicit blood coagulation reaction by facilitating physical crosslinking of blood component (Dowling et al., 2011; Rao et al., 2013). In their studies, researchers found that hMPs could aggregate with fibrin networks under a scanning electron microscope (SEM). So, they supposed that hydrophobic moieties on the particle surface might also interact with platelets and red blood cells besides fibrin networks, inducing a hemostatic effect of hMPs when they were sprayed to the esophageal wound. They also detected that these hMPs probably provided suitable scaffolds for growing of epithelial cells because of significant hydrophobicity and stiffness. In addition, the scaffold composed of hMPs was able to deliver and release vascular endothelial growth factor, which promoted angiogenesis (Yoshizawa et al., 2015). Moreover, the study discovered that the hydrogel layer transformed from hMPs in gastric ESD model of swine could suppress the fibrosis of gastric mucosa *via* interacting with cells, and ECM proteins multiply based on hydrophobic interactions. The number of inflammatory cells in the submucosa sprayed with hMPs obviously decreased compared to the control group. Meanwhile, the expression of α-smooth muscle actin (α-SMA) and type I collagen was significantly inhibited. Besides, the expression of von Willebrand factor (vWF) increased obviously in the hMP-treated group, indicating that the spraying of hMPs contributed to the growth and remodeling of blood vessels in the submucosa (Figure 6B). Above all, this sprayable wound dressing composed of naturally derived polymers exhibits multiple functions for wound healing based on hydrophobic interactions between polymers and biological tissues, establishing a good foundation for its clinical translation.

## Biomedical-Derived Materials

Biomedical-derived materials are originated from natural biological tissues that have undergone special treatments. Biological tissues may be taken from homologous or heterologous animal bodies. Special treatments include mild treatments such as fixation, sterilization, and eliminating antigenicity. Biomedical-derived materials have biological functions similar to those of natural organisms, and they play an important role in tissue repair. Therefore, biomedical-derived materials can be regarded as potentially ideal materials to be employed to prevent esophageal stricture.

### The Transplantation of Cell Sheets

**FIGURE 7:**
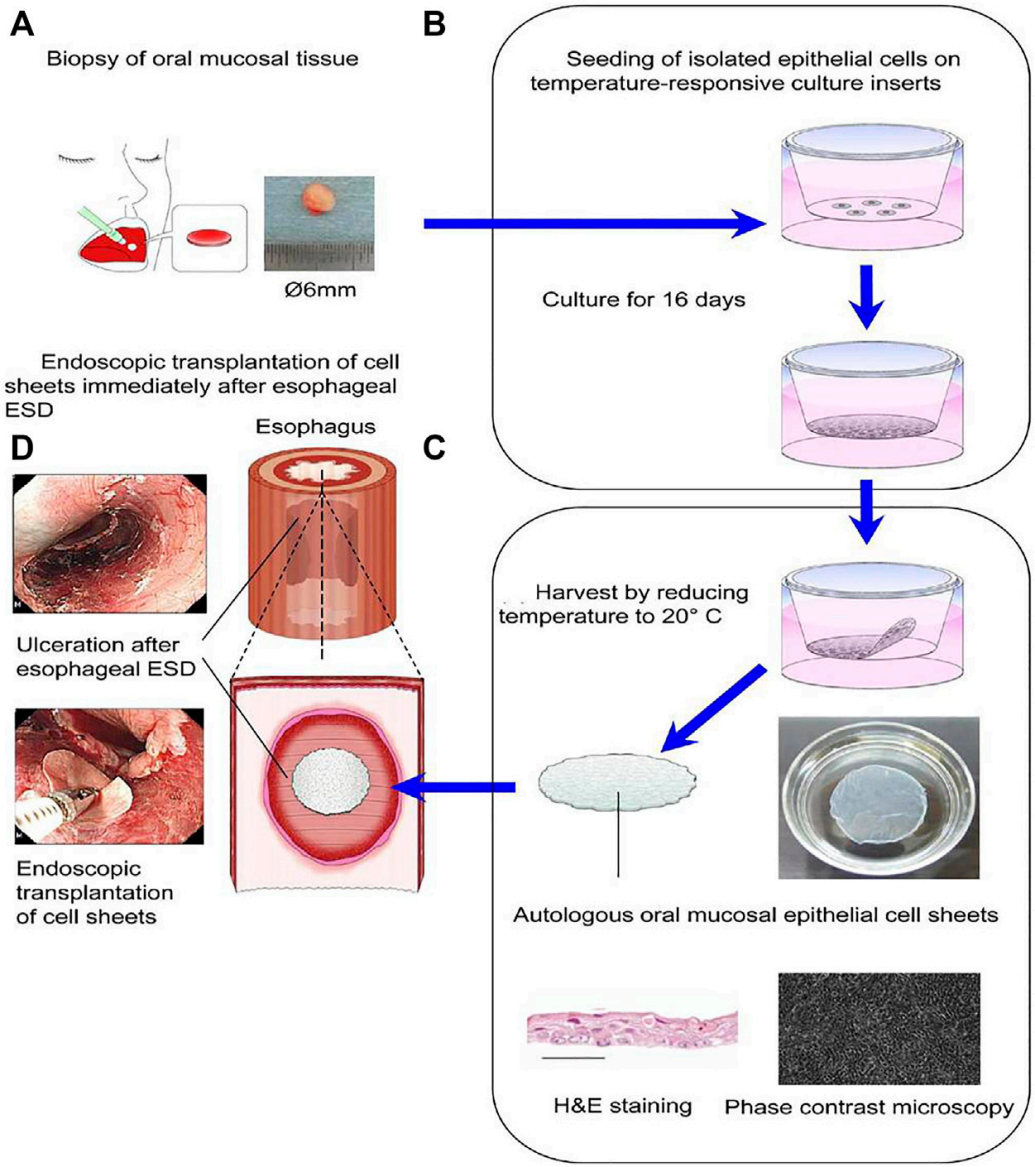
The treatment of the esophageal defect after ESD by transplantation of cell sheets composed of autologous oral mucosal epithelial cells. **(A)** Oral mucosal epithelial cells (OMECs) were taken from the patients’ oral cavity. **(B)** The OMECs were seeded on temperature-responsive culture inserts and cultured for 16 days. **(C)** Cell sheets composed of OMECs were harvested by reducing temperature to 20°C. **(D)** Cell sheets composed of OMECs were transplanted immediately on the esophageal defect under endoscopy after ESD. Reproduced from Ohki and Yamamoto (2020). Copyright (2020), with permission from Elsevier.

Cell sheet transplantation is a tissue engineering technology in which isolated cells are cultured at special conditions *in vitro*, retaining cell junctions and extracellular matrix in maximum. Specifically, after cells have been inoculated densely, cell sheet layers are formed by stimulating secretion of extracellular matrix, typically five to eight layers, and the intact extracellular matrix promotes cell growth. Cell sheets can act as barriers for defects to avoid being affected by food and other substances flowing through the esophagus. In the meantime, cell sheets may secrete various cytokines and growth factors to promote the proliferation of epithelial cells and wound repair. What is more, most cell sheets are originating from autologous cells, so there is no severe inflammatory reaction when they are applied to the surface of the wound. Cells and the extracellular matrix within the sheet can form interconnections with tissues through multiple pathways or interactions, enhancing adhesive strength between the cell sheet and the esophageal wound. As far as previous studies are concerned, cell sheets contain oral mucosal epithelial cell (OMEC) sheets (Ohki and Yamamoto 2020), compound cell sheets composed of OMECs and small intestinal submucosa (SIS) (Wei et al., 2009), and autologous skin epidermal cell sheets (Kanai et al., 2012). Ohki et al. (2012) discovered the promising potential of autologous OMEC transplantation to prevent esophageal stricture after ESD the earliest (Figure 7). Firstly, they harvested OMECs originating from patients’ oral cavity at normal condition in advance. Then, they treated cell sheets with temperature plunges to 20°C, and these cell sheets were transplanted to mucosal defects by endoscopy in patients immediately after ESD. After being transplanted to the defects, cell sheets adhered to the esophageal wounds, and they proliferated to form integral stratified epithelium. Wei et al. (2009) prepared the compound sheets composed of canine OMECs and porcine SIS, then they transplanted compound sheets on canine esophageal defects after ESD. After 1 month, the wounds treated by compound sheets completely repaired without inflammatory response. Additionally, the esophageal mucosal surface was as smooth as normal (Wei et al., 2009). Kanai et al. (2012) implanted cell sheets originating from porcine autologous skin epidermal cells into the post-ESD esophageal defect. As a comparison, another four pigs that underwent ESD without any treatments were given as control. After 2 weeks, the weight of pigs in the cell sheet group increased significantly compared to that in the control group, and there was no obvious esophageal stricture and inflammatory response (Kanai et al., 2012).

Above all, cell sheet technology seems indeed effective for preventing esophageal stricture after ESD by facilitating cell proliferation and promoting wound repair. Though reassuring advantages of cell sheets in preventing post-ESD esophageal stricture are shown, there are still some drawbacks and shortcomings. Firstly, the cell sheet technology has a high cost, and the preparation procedure *in vitro* is considerably complicated. Secondly, esophageal peristalsis and eating actions may cause cell sheets falling off, so how to anchor the transplanted cell sheets for a long time is a tough challenge. Thirdly, cell sheets must be prepared and preserved in a sterile condition; therefore, preserving cell sheets in a sterile environment for a long time is also a troublesome problem.

### Endoscopic Injection of Cell Suspension

**FIGURE 8:**
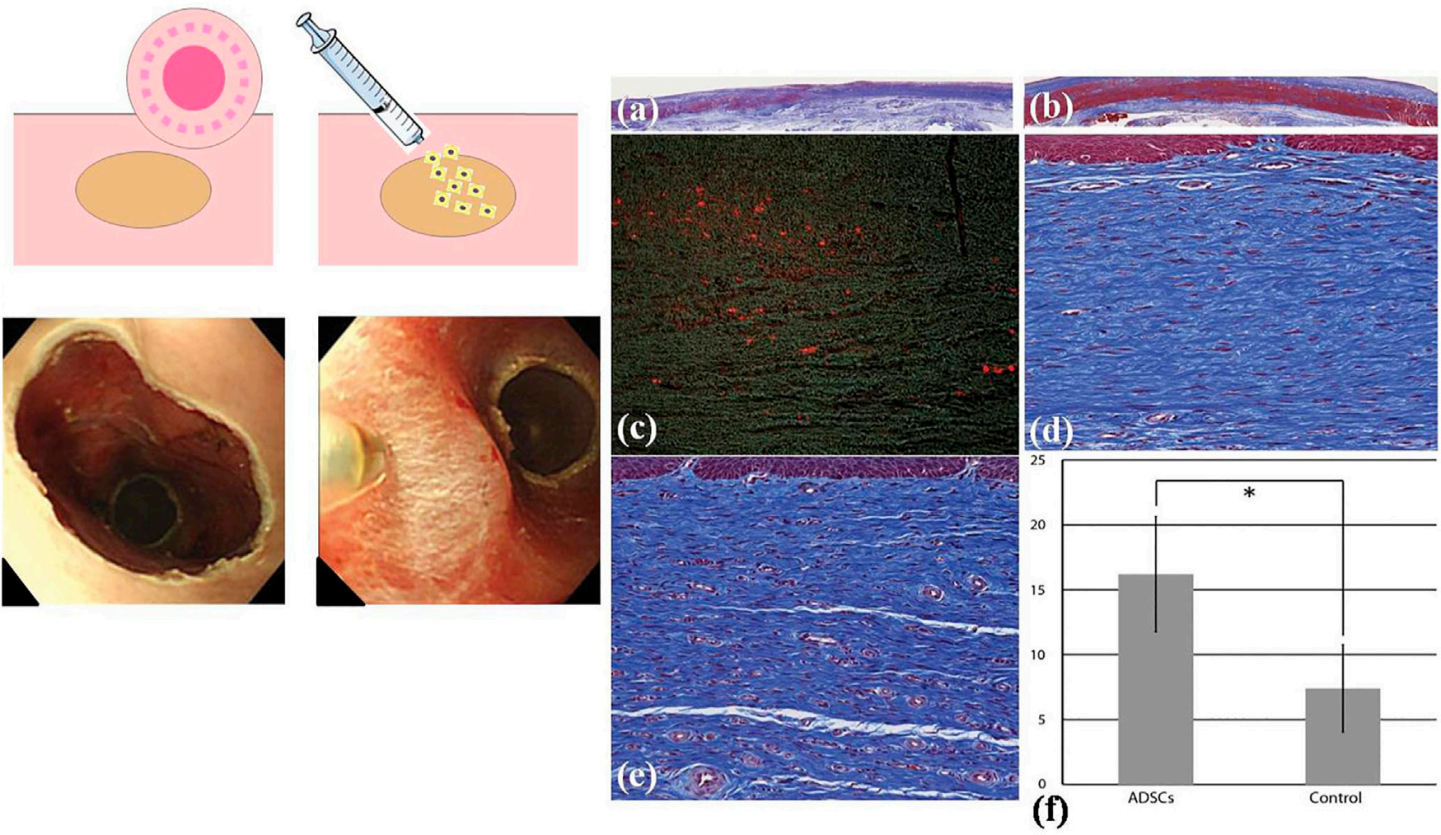
Adipose tissue-derived stromal cellADSC) injection to prevent esophageal stricture. **(A)** The schematic diagram of cell injection. **(B)** The esophageal defect left after EMR and ADSCs were injected into the submucosa of the esophagus. **(C)** The pathological analysis of the esophagus. **[C (i)]** The esophageal mucosa tissue in the control group (Masson trichrome). **[C (ii)]** The esophageal mucosa tissue in the ADSC-treated group. **[C (iii)]** Stain-labelled ADSCs (red color) in the submucosa of the esophagus in the cell injection group. **[C (iv)]** Submucosal layer of the esophagus in the control group. **[C (v)]** Submucosal layer of the esophagus in the ADSC-treated group. **[C (vi)]** The comparison of the number of microvessels in esophageal submucosa between the control group and the ADSC-injected group. Reproduced from Honda et al. (2011). Copyright (2011), with permission from Elsevier.

Owing to the relative simplicity, some scholars began to pay attention to cell suspension that can be injected endoscopically. During the process, autologous cells would be injected into defects (as illustrated in Figure 8), expecting to promote re-epithelialization and wound healing, so cell suspension can effectively prevent the occurrence of esophageal stricture in a way. The autologous cell suspension includes OMEC suspension, skin keratinocyte suspension, and adipose tissue-derived stromal cell (ADSC) suspension. Sakurai et al. (2007) injected autologous OMECs into the esophageal defects after ESD and achieved satisfying results. They made two postoperative defects in the esophagus of a pig. One was injected with autologous OMEC suspension into the submucosal layer immediately, and the other one was not treated. Two weeks later, there was no scarring stenosis in the injection group. The esophageal mucosa was smooth and the regenerating epithelium was completed. In the control group, there was scarring stenosis without the coverage of mucosal epithelium. Zuercher et al. (2013) reported that autologous skin keratinocyte suspension could prevent esophageal stricture effectively after EMR in sheep. Honda et al. (2011) used ADSCs to prevent esophageal stricture after EMR (Figure 8). They injected ADSC suspension into esophageal defects in five dogs under endoscopy after EMR (Figure 8A, B), and another five dogs were left untreated. After 2 months, serious esophageal stenosis occurred in the control group, while there was only a mild esophageal stricture in the injection group (Honda et al., 2011). The pathological findings of the esophagus showed that the mucosal layer of the esophagus was significantly damaged, and the submucosa was obviously fibrotic with few microvessels in the control group, while in the ADSC-injected group, the mucosa regenerated well, and there were more neovascularizations in the submucosa (Figure 8C). Above all, the effect of cell suspension injection has been partly displayed in some animal experiments. However, the mechanism of cell suspension injection has not been completely elucidated. There are two possible acting mechanisms: firstly, stratified epithelium may be formed owing to the proliferation of transplanted cells; secondly, transplanted cells may induce the secretion of cytokines and other nutrients needed by mucosal cells, promoting the migration of surrounding epithelial cells to the defect (Horch et al., 2005; Honda et al., 2011). Though cell suspension injection is simple and accessible without the need for lots of time and expenses, the limited isolated cells and low utilization need to be seriously considered. In addition, there is no research on the relationship between ADSCs and the remaining cancer cells, so it is still controversial whether injection of cell suspension into the wound will increase the risk of tumor recurrence, especially for the wounds with residual tumor cells.

### Extracellular Matrix Stents

**FIGURE 9:**
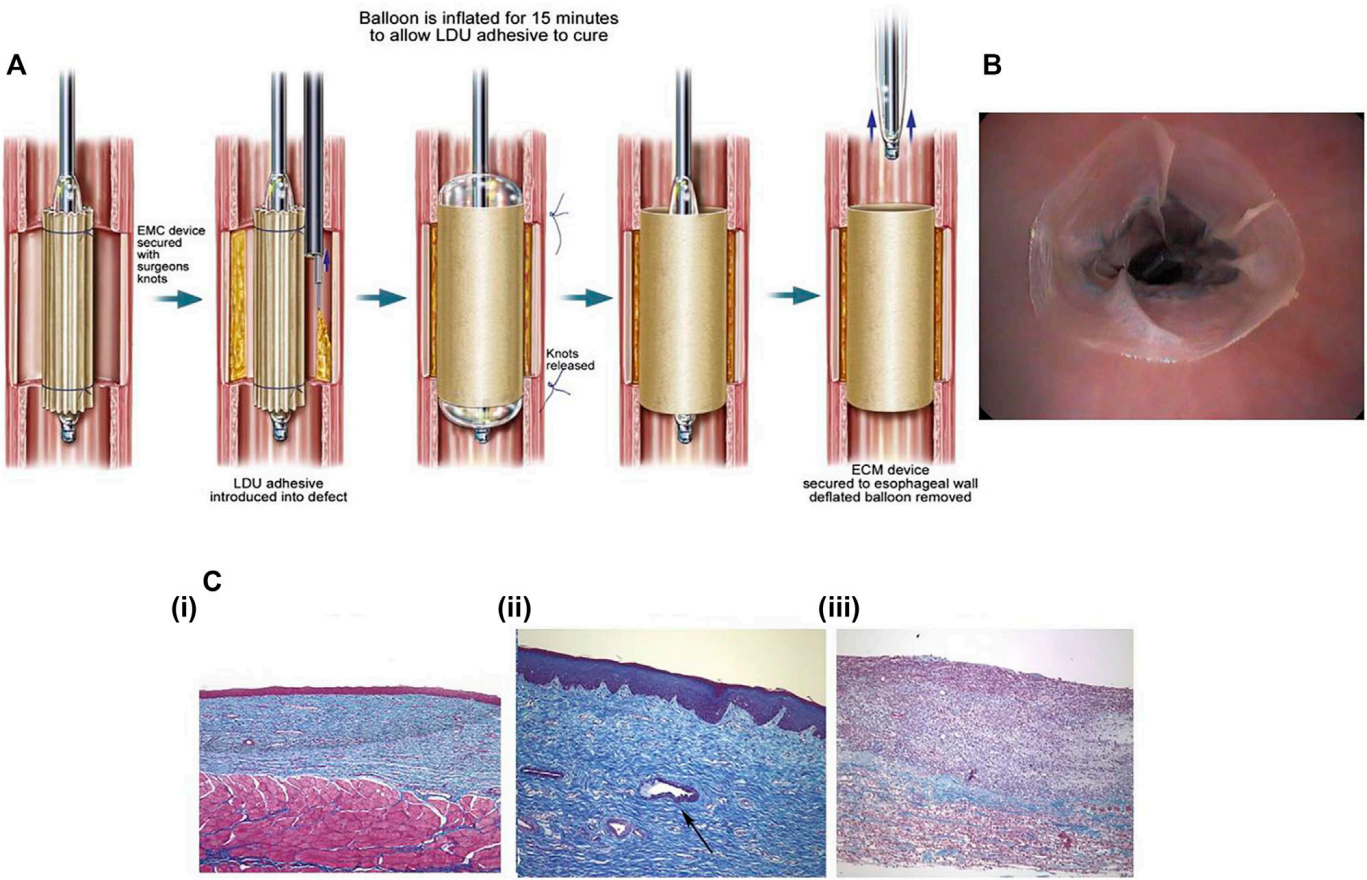
The ECM stents that have been reported to prevent esophageal stricture. **(A)** The schematic diagram of deployment of ECM stent with the internal supporting of balloons. **(B)** Endoscopic image of the ECM stent applied to post-EMR esophageal defect. **(C)** The histological assessment of the esophagus after 2 months of the deployment of ECM stents. **[C (i)]** The esophageal mucosa treated by ECM stents has intact squamous epithelium. **[C (ii)]** The gland formation in the submucosa of the esophagus treated with ECM stents. **[C (iii)]** The significant fibrosis with extensive infiltration of inflammatory cells in the esophageal submucosa in the control group. Reproduced from Nieponice et al. (2009). Copyright (2009), with permission from Elsevier.

The extracellular matrix (ECM) is a kind of macromolecule synthesized by cells and secreted outside the cells, and they are distributed on the surface of cells or between the cells. ECM mainly consist of polysaccharides, proteins, and proteoglycans. They can form complex grid structures spontaneously to support and connect cells. Besides, ECM contains a large number of signaling molecules that are actively involved in the control of cell growth, polarity, shape, migration, and metabolic activities. Attributed to the structure and biological functions of the ECM, they can be made into an artificial biological scaffold. The excellent physical and chemical properties of ECM make ECM stents have no pro-inflammatory effect and adapt well to biological tissues. A large number of cellular active components contained in ECM stents also promote tissue repair. Nieponice et al. (2009) applied ECM stents to prevent esophageal stricture after EMR in dogs (Figure 9). The study group treated with ECM stents had no presentation of esophageal stricture, while esophageal stricture occurred in the control group without implantation of ECM stents, accompanied by regenerated epithelium failing to cover mucosal defects with partial inflammatory response. However, there are few clinical trials at present, so there is a long and stiff road for ECM stents before being widely applied in clinical practice.

### Autologous Transplantation

With the aim of getting protection from physical barriers and promoting repair for esophageal wound, autologous esophageal mucosal transplantation has also been put forward, and it demonstrates a partial preventive effect in very limited clinical trials (Hochberger et al., 2014; Liao et al., 2018; Chai et al., 2019). In the process, researchers perform removal of normal esophageal mucosa and transplant it to the defect left by ESD. Hence, autologous esophageal mucosal transplantation is a tissue-shielding method, and it contains physical and biochemical factors of the native extracellular matrix. In certain aspects, autologous transplantation is similar to cell sheet. In summary, autologous esophageal mucosal patches can not only protect the esophageal wound as physical barriers but also promote angiogenesis and cell regeneration at the defect. Nevertheless, the corresponding clinical research sample of autologous transplantation needs to be expanded to study the feasibility, effectiveness, and safety further.

## Prospect of Biomedical Polymers and Potential Technology

Overall, many methods involving various biomedical materials have been introduced to prevent esophageal stricture after ESD, in which some have been widely used in the clinics and some are still under basic research. However, each method has its own advantages and limitations (Table 1). In clinical practice, steroid precautions, particularly systemic application, is currently one of the most common approaches to prevent post-ESD esophageal stricture. Tissue engineering technology demonstrates the prospect of restorative treatment. Autologous transplantation avoids immune rejection response. Other novel strategies like PGA sheets, CMC sheets, peptide hydrogels, and colloidal dressings have opened up new paths in preventing esophageal stricture based on the exploration of polymers.

**TABLE 1:** The advantages and limitations of strategies having been reported to prevent esophageal stricture after ESD.

	Approaches	Advantages	Limitations
Mechanical methods	Endoscopic balloon dilation	Sustaining long clinical use with quick effect	Demanding multiple dilatations; spending much time; uncomfortable to patients; and having the risk of bleeding, bacteremia, perforation, and re-stricture
	Metal stent implantation	Safe and effective to prevent esophageal stenosis	Prone to be displaced and having complications of bleeding and perforation
	Biodegradable stent implantation	No long-term complications, no need for manual removal, and can avoid re-injury of the esophagus	Poor capability of self-expansion, weak mechanical strength, and the placing process is complicated
Pharmaceutical prevention	Systemic steroid	Strong anti-inflammation and fibrosis-inhibition effect, very convenient, and accessible for patients to take	May induce many systematic side effects and lacking efficacy to esophageal mucosal defects
	Local injection of steroid	Powerful for inhibiting inflammation and decreasing the systematic side effects	Susceptible to complications related to injection operation
	Other medicine injections	Having an inhibitory effect on inflammation and fibrosis	Optimal dosage and usage time need to be clarified
Approaches involving biomedical polymers	PGA sheets	Successful to cover the defect and promote the regeneration of mucosal tissue	Difficult to stay at the defect for a long time, some patients may be allergic to them, and the feasibility and usefulness remain to be discussed for defects accompanied by bleeding
	Peptide hydrogels	Supporting the formation of functional and continued epithelial cell sheet	The study is performed on an *in vitro* model and how to utilize the hydrogel/dressing *in vivo* is to be discussed
	CMC sheets	Biocompatible and biodegradable and exhibit some preventive effects in several studies	Larger sample size and longer observation period are needed
	Colloidal dressing	Accelerating blood coagulation in defect, enhancing epithelialization, interacting with immune cells, and promoting angiogenesis	Experiments are done on animals at most currently, so safety and effectiveness are to be studied further
Methods covering biomedical-derived materials	Cell sheets	Promoting esophageal mucosal repair and inhibiting the degree of mucosal fibrosis	Accessible to falling off and having the risk of infection, huge costs, and the preparation process is cumbersome
	Autologous cell suspension	Simple and accessible to perform without requiring lots of time and economical expenses	Limited number of isolated cells and low utilization efficiency and having the risk of tumor recurrence
	Extracellular matrix stents	Having little pro-inflammatory effect, adapting well to esophageal defects, and containing a large number of cellular active components to promote tissue repair	There are few clinical trials
Autologous transplantation	Autologous gastro-esophageal mucosal/esophageal mucosal/skin transplantation	Having no inflammatory response and the grafting process is accessible	Lack of clinical samples

With the rapid development of biomedical polymers, several new approaches may be possible routes to prevent esophageal stricture. These polymers have ideal properties of biocompatibility and biodegradability to achieve their practicability. In addition, they can be combined with pharmacological prophylaxis and tissue engineering technology. Herein, we put forward two drug-loading (or other formulation) platforms: microneedles and hydrogel dressings. We study the practicality and feasibility of each system according to their inherent properties. Also, we analyze the application prospects after having discovered their strengths and weaknesses, respectively.

### Microneedle Technology

**FIGURE 10:**
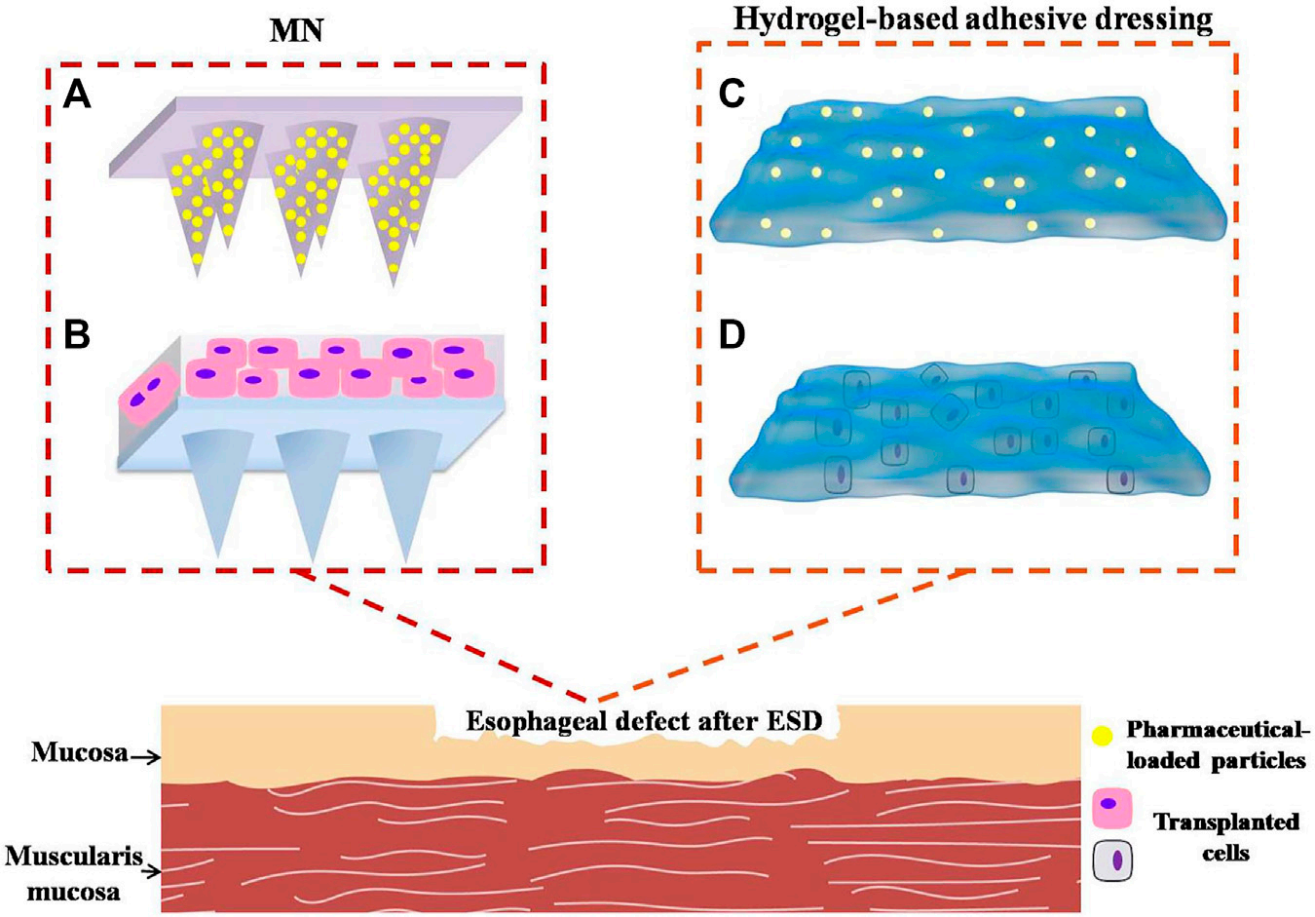
Novel potential strategies involve polymeric materials to prevent post-ESD esophageal stricture. **(A)** Microneedles loading anti-inflammatory or anti-fibrosis drugs into the needle body. **(B)** Microneedles incorporating active functional cells. **(C)** Hydrogel-based adhesive dressing loading pharmaceuticals. **(D)** Hydrogel adhesives incorporating transplanted cells.

Microneedles (MNs) have been utilized for medical treatment due to their ease of use over the past two decades. With their advantages of possessing capacities of injection and transdermal drug delivery, MNs have been proven to increase transdermal drug delivery efficiency significantly by penetrating though intrinsic tissue barriers in a minimally invasive manner, and they attract more and more attention from the medical field in recent years (Alexander et al., 2012). With the optimization of MN technology, non-transdermal MNs have demonstrated some effect in the management of diseases of the eyes, blood vessels, oral cavity, and mucosal tissues in animals (Lee et al., 2020). Lee et al. (2014) prepared a MN cuff (MNC) device to deliver anti-proliferation pharmaceuticals to vascular media and adventitia to prevent neointimal hyperplasia after grafting surgery. The MNCs were designed to wrap the exterior of the blood vessels, and the internal surface of MNCs contained an array of MNs, which is coated with drug formulation on tips. MNCs were installed on the blood vessels by embedding MNs, which were displayed onto the inner surface of MNC, into the walls of the blood vessels. Subsequently, the pharmaceuticals incorporated into the tips of MNs would be slowly and continuously released into the blood vessels, which guaranteed the sustained release and long-term effect of drugs. As a classical drug delivery system, MNs can load not only pharmaceuticals but also cells. Tang et al. (2018a) engineered a polymeric MN system integrated with cardiac stem/stromal cells (CSCs) for the treatment of myocardial infarction (MI). The MN-CSC system possesses certain superiorities compared with conventional patches. For instance, the MNs would serve as channels to allow for communications between CSCs in the patch and the host myocardium in heart tissues. The heart could provide CSCs in transplanted patch with nutrients. Meanwhile, CSCs in MN patch could release paracrine factors to repair myocardial tissues. Their study demonstrated that the MN-CSC patch brought bright prospect to facilitate effective treatment of MI by promoting angio-myogenesis and myocardial regeneration. After the treatment by MN-CSC, the myocardial functions gradually recovered. On the basis of the great application potential of non-transdermal MNs, the strategy of engineering polymeric MNs loading pharmaceuticals or other cytokines to prevent esophageal stricture can be put forward. The MNs can be attached to the surface of esophageal wound after ESD through mechanical interlocking (Figure 10A). Compared with ordinary needles, MNs have relatively soft insertions into the defects without sharp stimulations, which avoid serious complications such as perforation, bleeding, and other symptoms of the esophagus. This kind of MN-array system has two major functions, releasing drugs or cytokines to exert the appropriate pharmacological actions and forming a physical barrier like sheets to avoid irritation from food and other fluids passing through the esophagus.

In terms of drug release modalities, MNs can be divided into three types: burst release, prolonged release, and responsive release. According to the properties of anti-inflammatory drugs and duration of local inflammation reaction in esophageal wounds, the prolonged release and responsive release are preferred. Prolonged release means sustained delivery of pharmaceuticals, and this delivery mode helps to maintain a steady range of drug concentrations in the surrounding tissues for a considerable period. Generally, therapeutic agents can be incorporated into degradable microspheres or nanoparticles to realize the sustained release. For instance, Yang et al. (2019) made hydrogel-based MNs including the mesenchymal stem cell-derived exosomes and UK5099-loaded PLGA nanoparticles. The hydrogel-based MNs achieved a sustained release of UK5099 and exosomes for the duration of more than 10 days in a mouse model (Yang et al., 2019). The drug-loaded nanoparticles were evenly distributed within the bodies of MNs instead of just being localized on the surface of MNs. This engineering method could contribute to the slow release of the drug because of the gradual degradation of the polymeric matrices of MNs. Apart from this, the MN system can respond to the changes in the surrounding environment (including physical, chemical, and biological changes) to release the agents. By controlling the switch and intensity of the external stimulus, it is possible to control the release of pharmaceuticals in a timed, quantitative, and localized manner. Responsive delivery platforms are divided into closed-loop and open-loop systems (Jamaledin et al., 2020). Closed-loop control systems switch on and switch off drug release in a self-regulated manner to automatically achieve a circulating state, without any external intervention. For instance, glucose-responsive “closed-loop” insulin delivery systems mimicking the function of pancreatic cells possess a great potential to improve the health status and life qualities of people with diabetes (Ravaine et al., 2008; Bratlie et al., 2012). In their study, a glucose-monitoring module was combined with a sensor-triggered insulin-releasing module. A glucose-responsive insulin delivery strategy using a MN-array patch containing glucose-responsive vesicles (GRV) loaded with insulin and glucose oxidase enzyme was designed by some researchers. The components of GRVs contained a hydrophobic section, which transformed into hydrophilic composition under enzymatic hypoxic conditions. After that, GRVs dissociated and subsequently released the loaded insulin to lower blood glucose levels. If blood glucose was normal, significant enzymatic hypoxic conditions could not occur and hypoxia-sensitive GRVs would not dissociate to release insulin. In this way, precise regulation of blood glucose was guaranteed by GRV-loaded MN-array patch (Yu et al., 2015). Open-loop systems are known as an externally regulated-dependent platform (Kost and Langer 2001). They utilize external stimuli such as light, heat, magnetism, electricity, or mechanical stress to control loading release. Unlike closed-loop-responsive systems, stimuli are artificially added in the open-loop-responsive systems, and the stimuli will last for some time (such as light, magnetism, and heat). Closed-loop-responsive system allows for precise and rapid regulation of loading release, therefore, closed-loop-responsive MNs are frequently devised and studied.

When the MN patch is inserted on the surface of post-ESD defect under endoscope, pharmaceutical-loaded nanoparticles can be released into defect. The sustained release of pharmaceuticals will suppress inflammation or fibrosis. Polymeric MNs can degrade *in vivo* over time, eliminating the need for secondary endoscopic manipulation. Pharmaceuticals can also be released responsively to the inflammatory environment, which requires that the drug-loaded device should contain specific groups that are sensitive to inflammation-related enzymes or inflammatory factors. In the post-ESD esophageal environment, when the inflammation reaction is serious and local inflammatory mediators are numerous, the MN delivery system can sense the significant inflammation and release anti-inflammatory pharmaceuticals immediately to suppress the inflammation. When the inflammatory reaction becomes mild, MNs would correspondingly decrease the loading release. In such contexts, maximum efficacy and minimization of side effects can be realized. When inflammation is safely and effectively suppressed, the esophageal stricture would be further prevented.

What is more, esophageal mucosal epithelial cells, OMECs, ADSCs, autologous skin epidermal cells, and skin keratinocyte can be incorporated into the MN patch. These cells would proliferate and secrete some growth factors or other nutrients into defects to nourish mucosal cells and facilitate wound healing (Figure 10B).

Above all, with advances and optimizations of preparation processing, flexible, biocompatible, and degradable MN-array devices integrating sustained release or responsive release of drugs have a strong potential to prevent esophageal stricture. However, there is no report of MNs being used directly in the esophagus until now, which may be attributed to the special structure and functions of the esophagus. Apart from this, MN device is attached to the defect surface by mechanical cross-linking, so food passing through the esophagus may also affect the fixation of the MN system. Most importantly, a large number of studies are necessary to confirm the safety and biocompatibility of MNs in human bodies. Therefore, the selection of polymers to prepare a MN delivery system and the development of engineering technology demand comprehensive and in-depth explorations.

### Hydrogel-Based Wound Dressing

Hydrogels are soft materials possessing three-dimensional cross-linked network structures with flexible physical and chemical properties, which are similar to the natural ECM. In the recent decade, hydrogels have attracted worldwide attention, especially in drug delivery and tissue engineering. They have versatile characteristics: porous structures enable hydrogels to provide sufficient gas or nutrient exchange between the wound and the surrounding environment; a capacity to hold large amounts of water or biomedical fluids makes the hydrogel relatively comfortable for patients; good biocompatibility ensures safety; nice elasticity enables the hydrogel to be prepared with various shapes and sizes to conform to different wounds; and network structures render hydrogels able to reserve therapeutic nanoparticles and other biomedical reagents within them (Huang et al., 2017; Koehler et al., 2018; Li et al., 2020). In addition, the degradability of hydrogels reduces the pain of patients and avoids secondary tissue damages (Stern and Cui 2019). In recent years, hydrogel-based dressings have been developed to meet the requirements of wound healing because of the strong adhesion besides the advantages of hydrogels mentioned above. In some wounds accompanied with a high level of inflammatory exudate, hydrogel-based dressings can absorb exudates to facilitate the debridement process. In general, hydrogels composed of biocompatible and biodegradable biomedical materials can be optimized into ideal wound dressings. A variety of widespread biomedical polymers have been employed to engineer hydrogel-based dressings, such as chitosan, hyaluronic acid (HA), gelatin, polyethylene glycol, alginate, and several other less-common polymers (Chen et al., 2018; Dimatteo et al., 2018; Koehler et al., 2018; Qu et al., 2018).

Enlightened by the adhesion phenomena of natural organisms, researchers have developed hydrogels with different adhesion mechanisms for wound dressings. For instance, marine mussels can form strong adhesions to wet surfaces through mussel foot protein (mfp) secreted by foot filaments. The main component of mfp is L-DOPA (3,4-dihydroxyphenylalanine), which is rich in catechol structures (Ahn et al., 2015). By means of imitating specific features of marine mussels, the mfp-like adhesion component can be introduced into hydrogels *via* grafting catechol groups into the polymer chains. These catechol groups would bring many physical and chemical interaction forces to make hydrogel dressings adhere strongly to tissue surfaces, including van der Waals forces, metal chelation, hydrogen bonds, π-π stacking, Schiff base reaction, and Michael addition reaction (Burzio and Waite 2000; Li et al., 2010; Wang et al., 2017; Zhou et al., 2020). Lee et al. (2010) fabricated injectable and thermo-sensitive hydrogels based on HA and Pluronic through using rapid and robust catechol-thiol reactions. In study, HA conjugated with dopamine was mixed with thiol-bonded Pluronic F127 copolymer to produce a cross-linked composite gel based on typical Michael addition reaction. The HA/Pluronic hydrogels could be injected in a sol state at room temperature, but immediately turn into a robust gel at body temperature. The composite gel exhibited excellent tissue-adhesion capacities with superior stability *in vivo*. Moreover, the catechol-based adhesives have been proven to possess good dehydration effect, and they can form strong adhesion on wet tissues (Wei et al., 2016).

Besides using polymers containing the catechol group, other chemical groups are equally capable to build covalent bondings between hydrogels and the amine groups on tissue surfaces. Qu et al. (2018) synthesized an injectable hydrogel adhesive composed of quaternized chitosan and benzaldehyde-terminated Pluronic^®^F127 (PF127-CHO) under physiological conditions, in which a Schiff base was constructed between the aldehyde group in PF127-CHO and the amine group on the tissue surface, so the hydrogel adhesive could adhere to the skin tissues. Bu et al. (2019) prepared a clearly defined tetra-armed poly-(ethylene glycol) (Tetra-PEG) hydrogel sealant *via* ammonolysis. The sealant owned competencies of fast gelating speed, strong tissue adhesion, and high mechanical strength. The active ester of succinimidyl succinate on the polymer chain reacted with the amino group on tissues and made the hydrogel attach to the skin. Some other adhesion mechanisms such as topological adhesion and electrostatic interaction also have a good adhesion effect, though the preparation process should be further improved to avoid toxicity (Cho et al., 2019; Krishnadoss et al., 2019). Khalil et al. (2020) prepared an antibacterial adhesive hydrogel loaded with micelles containing ciprofloxacin (CPX) for the treatment of corneal injuries with risk of infection. The results showed that the loading of CPX did not affect the stiffness, biocompatibility, and adhesive strength of hydrogels, suggesting a potential solution to seal corneal wounds without being infected. You et al. (2021) designed poly (2-ethyl-2-oxazoline-co-2-butenyl-2-oxazoline) (POx) hydrogels encapsulating mesenchymal stromal cells (MSCs). The thiol-ene crosslinked hydrogels exhibited great tissue adhesive strength. In a rat model of myocardial infarction, the epicardial placement of MSC-loaded POx hydrogels promoted the recovery of cardiac structure and functions with decreased interstitial fibrosis and increased formation of neovasculars. All these studies demonstrated that hydrogel adhesives engineered by a variety of natural or synthetic polymers can be utilized for effective delivery of drugs and cells.

As for wet esophageal defect after ESD, the hydrogel adhesives are expected to play dramatic roles in the prevention of esophageal stricture. Firstly, hydrogel adhesive can be adhered to the esophageal wound firmly and tightly owing to the powerful adhesions originating from different mechanisms. Secondly, they possess ideal physical and chemical properties to support defects and promote wound healing. Thirdly, steroids or anti-fibrotic drugs can be loaded into the hydrogel adhesive in the form of drug carriers (Figure 10C) to achieve sustained release of pharmaceuticals, such as micelles, vesicles, or other nanoparticles. OMECs, ADSCs, or other skin keratinocytes can also be incorporated into hydrogel adhesives to facilitate mucosal repairing and epithelialization (Figure 10D). Above all, the hydrogel adhesive has a great potential to be applied to manage post-ESD defect, despite the compatibility and biodegradability needing further improvement. In prospect, we expect to observe the safety and efficacy of hydrogel adhesives for the prevention of esophageal strictures in more animal studies and preclinical trials.

## Summary and Conclusion

Esophageal stricture is a common and serious post-ESD complication, but until now, there is no standard consensus to effectively manage it. In clinical practice, preventing this complication in advance improves the patients’ prognosis and life qualities compared to intervening after it has developed. However, these methods often bring inevitable and serious complications such as bleeding, esophageal perforation, tissue adhesion, and other related symptoms. In this review, we have summarized the preventive strategies of esophageal stricture involving biomedical materials that have been reported in recent decades. Firstly, we have described the mechanism of the formation of esophageal stricture; secondly, we introduced novel preventive methods covering biomedical materials; thirdly, we put forward some prospective strategies related to biomedical polymers. Generally, the basic requirements for biomedical materials include good biocompatibility, biodegradability, safety, and stable chemical properties. In addition to these features mentioned above, some biomedical polymers and biomedical-derived materials possess network structures similar to those of natural ECM, providing scaffolds that facilitate cell adhesion, proliferation, and differentiation. Besides, they have good mechanical properties, matching the structural mechanics of the natural tissues. Additionally, some biomedical materials have superior surface physico-chemical properties to form specific adhesion on tissue surfaces, and they can load functional biological agents. The approaches employing these biomedical materials demonstrate some preventive effect and safety in post-ESD esophageal stricture. In particular, PGA sheets and CMC sheets have been studied in a very small number of clinical patients. Therefore, the safety and feasibility of biomedical polymers need to be explored further. Other prospective technologies engineered by various natural or synthetic polymers, like chitosan, hyaluronic acid, and so on, also require systematic and comprehensive observational studies *in vitro* and *in vivo* before being performed into clinical trials. Above all, biomedical materials provide important and far-reaching inspiration for the prevention of esophageal strictures after ESD, especially biomedical polymers and biomedical-derived materials. Nevertheless, there are some non-negligible bottlenecks for their clinical applications, such as specific immunogenicity, sensitization to patients, slightly poor histocompatibility, and incomplete degradability. All these issues are anticipated to be resolved basically in the near future. We believe that biomedical materials are promising to play a pivotal role to prevent esophageal stricture after ESD safely and effectively.
